# Terbinafine Nanohybrid: Proposing a Hydrogel Carrying Nanoparticles for Topical Release

**DOI:** 10.3390/pharmaceutics15030841

**Published:** 2023-03-04

**Authors:** Louise Lacalendola Tundisi, Janaína Artem Ataide, Jéssica Heline Lopes da Fonseca, Luiza Aparecida Luna Silvério, Marcelo Lancellotti, Ana Cláudia Paiva-Santos, Marcos Akira d’Ávila, Daniel S. Kohane, Priscila Gava Mazzola

**Affiliations:** 1 Faculty of Pharmaceutical Sciences, University of Campinas (Unicamp), Campinas 13083-871, SP, Brazil; 2 Laboratory for Biomaterials and Drug Delivery, Department of Anaesthesiology, Division of Critical Care Medicine, Boston Children’s Hospital, Harvard Medical School, Boston, MA 02115, USA; 3 Department of Manufacturing and Materials Engineering, School of Mechanical Engineering, University of Campinas (Unicamp), Campinas 13083-860, SP, Brazil; 4 Department of Pharmaceutical Technology, Faculty of Pharmacy of the University of Coimbra, University of Coimbra, 3000-548 Coimbra, Portugal; 5 REQUIMTE/LAQV, Group of Pharmaceutical Technology, Faculty of Pharmacy of the University of Coimbra, University of Coimbra, 3000-548 Coimbra, Portugal

**Keywords:** hydrogel, terbinafine, poloxamer 407, nanoparticle, drug release, rheology, polycaprolactone

## Abstract

A poloxamer 407 (P407)—Casein hydrogel was chosen to carry polycaprolactone nanoparticles carrying terbinafine (PCL-TBH-NP). In this study, terbinafine hydrochloride (TBH) was encapsulated into polycaprolactone (PCL) nanoparticles, which were further incorporated into a poloxamer-casein hydrogel in a different addition order to evaluate the effect of gel formation. Nanoparticles were prepared by the nanoprecipitation technique and characterized by evaluating their physicochemical characteristics and morphology. The nanoparticles had a mean diameter of 196.7 ± 0.7 nm, PDI of 0.07, negative ζ potential (−0.713 mV), high encapsulation efficiency (>98%), and did not show cytotoxic effects in primary human keratinocytes. PCL-NP modulated terbinafine was released in artificial sweat. Rheological properties were analyzed by temperature sweep tests at different addition orders of nanoparticles into hydrogel formation. The rheological behavior of nanohybrid hydrogels showed the influence of TBH-PCL nanoparticles addition in the mechanical properties of the hydrogel and a long-term release of the nanoparticles from it.

## 1. Introduction

Terbinafine (TBH) is an antifungal agent that is indicated for the treatment of dermatophyte infections. This agent acts by inhibiting the synthesis of ergosterol, and is available in the form of tablets, creams, sprays and gels [1]. Despite being available in several formulations, TBH has some drawbacks, including hepatic and renal toxicity, and has potential drug interactions. Topical therapy with TBH shows many advantages over oral use, such as no first-pass effect, less drug interactions, high local drug concentration, laboratory monitoring during treatment is usually not required, it is considered a non-invasive route, and it reduces systemic toxicity [2,3,4]. However, TBH topical treatment of ringworm, for example, is slow and uncomfortable, requiring multiple applications per day, during a period that can vary from 2 weeks to more than a year [1,5], impairing the patient’s compliance. Unfortunately, TBH is only one example of many other drugs that could benefit from the developing of drug delivery systems to achieve a more controlled and effective delivery, helping patients through their treatments [6,7,8].

Those efforts in improving treatments stimulates the search for advances in new polymeric materials and the development of drug delivery systems. Hydrogels are tridimensional polymeric networks that have been extensively studied, mainly for topical applications [9]. A poloxamer-casein hydrogel was recently developed as an option for local long-term treatment, providing modified release and adhesiveness [4]. Despite the advances in this area, several challenges still exist, and to overcome them, hybrid systems carrying nanoparticles have been developed [10].

Polymeric nanoparticles are generally suitable for topical drug delivery once they tend to accumulate in the skin compared to solid lipid nanoparticles and carriers of lipid nanostructures [11,12], reducing permeation to deeper layers [13,14], avoiding systematic off target delivery, and sticking to the local delivery. When embedding the nanoparticles in a pharmaceutical formulation such as hydrogel, other characteristics and/or advantages in the treatment and in the product’s organoleptic characteristics can be achieved [7,8]. These nanohybrid hydrogels can bring unique physicochemical and mechanical properties that cannot be reached in single-component systems, and they also show multi-functionality [15].

In this study, we describe the development of a nanohybrid composed of poloxamer-casein hydrogel embedding polycaprolactone (PCL) nanoparticles loaded with TBH. The novelty in this system is the presence of two drug delivery vehicles that can provide a greater control of drug release and enhance the residency of nanoparticles into the skin. To this end, PCL nanoparticles containing TBH were produced by the nanoprecipitation technique and further dispersed into poloxamer-based hydrogels. Here we report the characterization of NPs (mean diameter, polydispersity index (PDI), zeta potential (ζ potential), attenuated total reflectance, Fourier transform infrared (ATR-FTIR), morphology, encapsulation efficiency, drug release profile and cytotoxicity), as well as the nanohybrid hydrogel rheological properties and particle release.

## 2. Materials and Methods

### 2.1. Materials

Poloxamer 407 (P407, pharmaceutical grade) was kindly donated from BASF (Florham Park, NJ, USA), casein (powder with 90% protein content) and polycaprolactone (PCL, average molecular weight 80,000) were purchased from Sigma-Aldrich (Atlanta, GA, USA), PCL-fluorescein (>90% PCL 80,000 MW and fluorescein functionality) was purchased from APM polymer (Montreal, QC, Canada), and terbinafine hydrochloride (pharmaceutical grade) was purchased from Frontier Scientific Inc. (Logan, UT, USA). All other reagents were of analytical grade.

### 2.2. PCL-Terbinafine Nanoparticle Formulation

Terbinafine PCL nanoparticles (NP-TBH-PCL) were produced by nanoprecipitation. Polymer samples were previously dissolved in acetone to reach a concentration of 2.5 mg/mL, along with given amounts of terbinafine (0.500 mg, representing 20% *w*/*w*). The resulting organic solutions were stirred at 60 °C until polymer dissolution. The formation of nanoparticles was then induced by fast adding 12.5 mL of the organic phase into 50.0 mL of P407 solution 2.5% (*w*/*w*) prepared earlier using Milli-Q^®^ water. The stirring speed of the aqueous phase was kept fixed (350 rpm). The resulting solutions were stirred at 37 °C overnight to allow for solvent evaporation. The assay was carried out in triplicate. The final aqueous volume after the nanoprecipitation procedure and organic solvent elimination was set to 50.0 mL. Blank nanoparticles (NP-PCL) were prepared similarly, excluding the addition of terbinafine.

### 2.3. Nanoparticle Characterization

#### 2.3.1. Nanoparticle Size Distribution and Zeta Potential

Particle size, polydispersity index (PDI) and zeta potential of NP-TBH-PCL and NP-PCL formulations previously diluted in Milli-Q^®^ water (1:4, *v*/*v*) were determined via the dynamic light-scattering procedure in Zetasizer Nano ZS (Malvern Instruments, Malvern, UK). Particle size and zeta potential were measured in triplicate at room temperature.

#### 2.3.2. Transmission Electron Microscopy (TEM)

The morphology of particles was observed by a transmission electron microscope (TEM) (Tecnai G2 Spirit BioTWIN, FEI, Hillsboro, OR, USA). Carbon film 300 mesh was sunk into the particle suspension (liquid after organic solvent evaporation), dried with filter paper, and stained with urinol acetate saturated (UAS) for TEM analyses. Images were acquired using 23 kX magnification.

#### 2.3.3. Terbinafine Encapsulation Efficiency (EE)

Encapsulation efficiency (EE%) was determined by the indirect method, as the difference between total terbinafine in NP-TBH-PCL and non-encapsulated terbinafine, using Equation (1). To determine total terbinafine in NP-TBH-PCL, the whole NP suspension was lysed by adding acetonitrile (1:20) and stirring for 30 min. Cold methanol (1:3) was added and stirred again for 30 min. After that, the solution was ultra-centrifuged at 25,000× *g* for 30 min and the supernatant was collected and analyzed by a spectrophotometer at 283 nm. Non-encapsulated and encapsulated terbinafine were separated by two-step centrifugation: first, the NP suspension was centrifuged at 25,000× *g* for 30 min; the supernatant was then collected and centrifuged using Amicon Ultra-0.5 mL centrifugal filters (10 kDa MWCO) at 14,000× *g* for 15 min. The filtrate was collected and analyzed for terbinafine by spectrophotometer UV (Multiscan GO, Thermo Scientific, Waltham, MA, USA) at 283 nm. Quantifications were always performed in triplicate.

(1) $$\mathrm{EE}\left(\%\right)=\frac{\mathrm{Total}\;\left(µg\right)-\mathrm{Non-encapsulated}\;\left(µg\right)}{\mathrm{Total}\;\left(µg\right)}\times 100$$

#### 2.3.4. Fourier Transform Infrared (FTIR)

The FTIR spectra of NP-TBH-PCL were recorded in an ATR-FTIR (attenuated total reflectance Fourier transform infrared) spectrometer (Nicolet iS50, Thermo Fisher Scientific, Waltham, MA, USA) operating between 4000 to 600 cm^−1^. Nanoparticles were analyzed in suspension, while the other compounds (PCL, TBH and P407) were analyzed in their solid state.

#### 2.3.5. Terbinafine Release from Nanoparticle Formulation

The in vitro release profile of TBH from nanoparticles was evaluated using the vertical diffusion Franz cell system with a diffusion area of 0.64 cm^2^. Regenerated cellulose dialysis membranes (14 kDa MWCO) were placed between the donor and receptor cells and subsequently clamped. The receptor cell was filled with pre-warmed artificial sweat pH 4.7 (20 g/L sodium chloride, 18 g/L ammonium chloride, 5 g/L acetic acid, 15 g/L lactic acid adjusting pH to 4.7 with NaOH 3M) and the donor cell with 1 mL of NP-TBH-PCL or free-TBH solution for comparison (sextuplicate for each sample). The system was kept under 37 °C and at pre-determined intervals 0.3 mL was taken from the receptor compartment for TBH quantification at 283 nm in spectrophotometer UV. The withdrawn volume was replaced with pre-warmed receptor medium right after each sampling. The permeation of TBH across membrane (%) was calculated considering the initial amount of TBH placed in the donor compartment, following Equation (2):

(2) $$\mathrm{TBH}\;\mathrm{Release}\;\left(\%\right)=\frac{\mathrm{TBH}\;\mathrm{cumulative}\;\mathrm{release}\;\left(\mu g\right)}{\mathrm{TBH}\;\mathrm{initial}\;\left(\mu g\right)}\times 100$$

Steady-state permeation flux (Jss, µg/cm^2^/h) was determined following Equation (3):

(3) $$\mathrm{Jss}={\left(\frac{\mathrm{dQ}}{\mathrm{dt}}\right)}_{\mathrm{ss}}\times \frac{1}{A}$$

where, (dQ/dt)ss is the amount of TBH (µg) permeated through time (h), and A is the diffusion area (cm^2^).

The data from the TBH release profile from free-drug solution and nanoformulation were kinetically evaluated using various mathematical models, such as zero order, first order, Higuchi and Korsmeyer-Peppas model equations [16]. The best mathematical fit was chosen by comparing models using the extra sum-of-squares F test.

#### 2.3.6. Nanoparticle Cytotoxicity

The cell viability was performed with the HaCat (keratinocytes) cell line, and cultured in RPMI media with 10% fetal bovine serum. Cells were plated approximately 5.4 × 10^4^ cells/well in 24 well plates, incubated at 37 °C and 5% CO_2_, after 24 h and total cell adherence. Cells were washed in phosphate saline buffer (PBS), and then exposed to 312.5; 156.25; 39.06; 19.53; 9.76; 4.88 and 2.44 μg/mL of NPs (blank and loaded with terbinafine) diluted in RPMI media, respectively. The cells were exposed for 24 and 48 h in incubation at 37 °C and 5% CO_2_.

After treatment, the culture medium was removed and 500 μL culture medium with 100 μL MTS (3-(4,5-dimethylthiazol-2-yl)-5-(3-carboxymethoxyphenyl)-2-(4-sulfophenyl)-2H-tetrazolium bromide) was applied to the culture, which was incubated for 2 h at 37 °C and 5% CO_2_. The solution was then withdrawn and placed in 96-well plates and the absorbance was read at 570 nm (Readwell Touch, Robonik, India). All tests were performed in quadruplicate. Cell viability was calculated as the percentage of non-treated cells (RPMI media only) as a control.

### 2.4. P407-Casein Hydrogel Formulation

The hydrogels were prepared as previously described [4]. In general, casein solution (50 mg/mL) was prepared by dissolving casein in deionized water and the pH was adjusted to 7 using a 5 M NaOH solution. The obtained casein solution was stored in a 4 °C refrigerator. P407 (18%, *w*/*v*) was added and the solutions were stirred overnight at 4 °C for gel formation [4]. Five different concentrations (0.2; 0.4; 0.8; 1.6 and 2 mg/mL) of NP-TBH-PCL were added to the hydrogel for rheological and texture analysis. This addition was done in two different orders. M1: Casein solution, P407, nanoparticles. M2: Casein solution, nanoparticle, P407.

#### 2.4.1. Rheological Analysis

Rheological characterization was performed with P407 18% Casein 10% hydrogel at different concentrations of the NP-TBH-PCL to analyze the nanoparticle influence in the rheological behavior. Five different concentrations (0.2; 0.4; 0.8; 1.6 and 2 mg/mL) of NP-TBH-PCL were added to the hydrogel. This addition was done in two different orders. M1: Casein solution, P407, nanoparticles. M2: Casein solution, nanoparticle, P407. Hydrogel’s rheological analysis were carried out on a Modular Compact Rheometer (MCR-102, Anton Paar, Graz, Austria) using a plate-plate geometry. The plate diameter was 50 mm with a gap of 0.3 mm. Temperature sweep assays were performed at a temperature range from 10 °C to 45 °C at a heating rate of 1 °C/min, angular frequency of 100 rad/s, and strain of 1% in the linear regime. Experiments were performed in triplicate. Gelation temperature was taken as the temperature at which G′ becomes greater than G″.

#### 2.4.2. Thixotropy Analysis

Thixotropic behavior was analyzed by hysteresis loop tests. Hysteresis loop areas were obtained with a consecutive increased shear rate from 1 to 1000 s^−1^ (upward curve) during 165 s, kept for 50 s (plateau), and followed by a gradual decrease to 1 s^−1^ (downward curve). The analysis was performed at room temperature (25 °C) and 37 °C.

#### 2.4.3. Texture Analysis

The spreadability analysis was performed using a texturometer (Stable Micro Systems TA.XT plus, Godalming, UK) using compression mode. The analyses were performed according to the pre-established parameters shown in Table 1. The formulations were placed in the female cone and pressed down to eliminate air pockets. Firmness (g) and work of shear (g/s) were calculated. The tests were performed in triplicate.

#### 2.4.4. Particle Release from P407-Casein Hydrogel

We used the same method as that used in Section 2.2 but with PCL linked fluorescein (PCL-fluorescein) instead of regular PCL. A concentrated nanoparticle solution was added to the formulation (NP-PCL and NP-TBH-PCL) and stirred until complete dispersion. Using Yang, et al.’s [17] adapted method, transwell membrane inserts (0.4 mm pore size and 0.33 cm^2^ area; Costar) and 24-well culture plates were used as the donor and acceptor chambers, respectively. Two hundred microliters of each formulation were pipetted directly onto pre-warmed filter inserts to obtain a solid hydrogel. Filter inserts (donor compartments) with formed gels were suspended in wells (acceptor compartments) filled with pre-warmed artificial sweat at a pH of 4.7, and the plates were then incubated in an oven (37 °C). At each sampling time (0.5, 1, 2, 6, 12, 24, 48 h), 1 mL aliquots of the artificial sweat-receiving media were collected, and inserts were sequentially moved into a new well with fresh media. Sample aliquots were analyzed by fluorescence spectroscopy (Sinergy HT, Bio-Tek Instruments, Winooski, VT) (λ_ex._ 490 nm and λ_em._ 514 nm). The same amount of concentrated nanoparticle solution added to the hydrogel was measured, and its fluorescence was set as 100%. Experiments were performed in quadruplicate. The data from the NP release profile from hydrogel formulation was also kinetically evaluated using mathematical models [16].

### 2.5. Statistical Analysis

Data are expressed as means ± standard deviations. All statistical comparisons were made with a Student’s *t*-test, unpaired for independent samples. A *p* value < 0.05 was considered to indicate statistical significance. For mathematical model fitting, Prism 5 software (GraphPad Software Inc., San Diego, CA, USA) for Windows was used.

## 3. Results and Discussion

### 3.1. Nanoparticle Characterization

The particles were produced using P407 (Poloxamer P407) as stabilizers in the aqueous phase, aiming for their future incorporation into the hydrogel formulation. Terbinafine hydrochloride is a very suitable antifungal agent for the treatment of dermatophyte infections. It is a highly lipophilic active that tends to accumulate in skin, nails, and adipose tissue [18]. Blank nanoparticles (NP-PCL) and nanoparticles carrying terbinafine (NP-TBH-PCL) were successfully produced and characterized by dynamic light scattering (DLS). NPs showed a significant difference in size of approximately 10 nm (*p* < 0.0001). We can consider the nanoparticle solution as monodisperse, as both particles presented a low polydispersity index (PDI), and NP-TBH-PCL presented a significant reduction (*p* < 0.05) in this parameter (Table 2). In both cases, the Zeta potential was as low as P407, a non-charged polymer, was used as a steric hindrance stabilizer for the nanoparticle solution. However, the Zeta potential of NP-TBH-PCL was significantly higher than NP-PCL (*p* < 0.0001).

**Figure 1:**
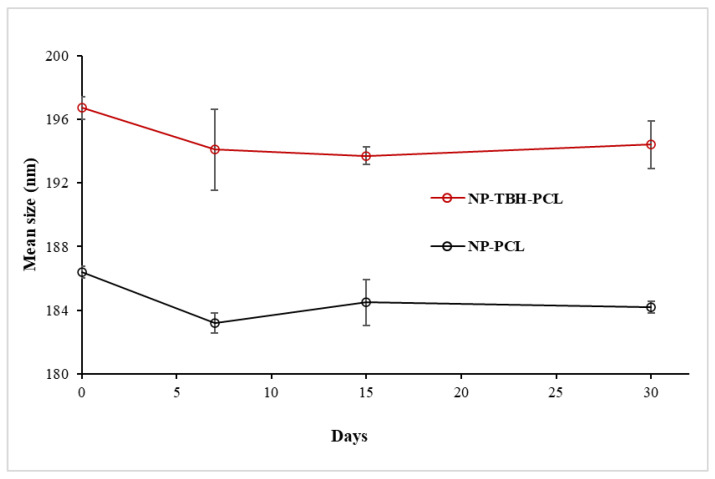
Stability study of the nanoparticle mean size (nm) over a month. Measures were taken at days 0, 7, 15 and 30. Results are shown as average ± SD. Some error bars are obscured by the symbols due to their small size.

Even though it is very common to relate Zeta potential equal or higher than ±30 mV to stable nanosystems, a broad discussion is showing that the physical stability of nanoparticles depends not only on electrostatic, but also on steric, entropic, and Van der Waals forces [19]. The addition of non-ionic surfactants and the resulting steric effect has already been used to explain physical stable nanosuspensions with a Zeta potential below ±20 mV [20,21]. Poloxamer P407, a non-ionic surfactant, was used in the PCL-NP formulation previously [22,23]. In those cases, even though the Zeta potential was lower than ±10 mV, the NP was stable due to the steric stabilization given by P407 distribution around NP’s surface. To assess the samples’ physical stability, a one-month stability study was performed with both particles, and no significant difference (*p* > 0.05) in mean size was observed over time (Figure 1).

**Figure 2:**
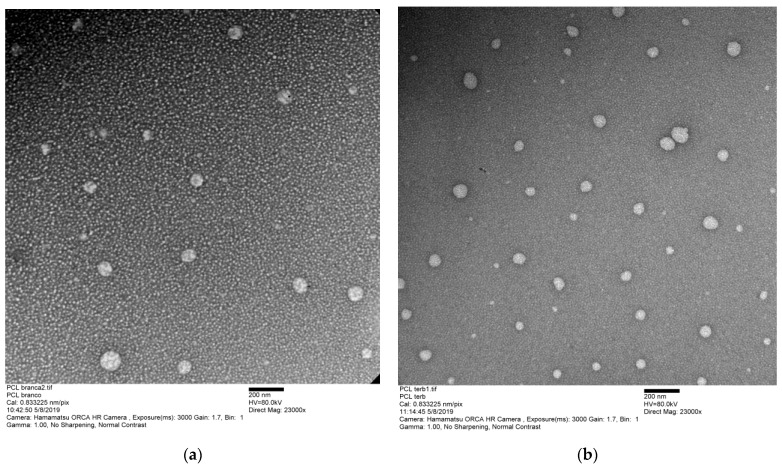
Transmission electron microscopy (TEM) image of PCL nanoparticles (**a**) NP-PCL and (**b**) NP-TBH-PCL in P407 2.5% solution.

The particle morphology was analyzed by transmission electron microscopy (TEM, Figure 2), the mean size of the blank nanoparticle was 89.42 nm, and the terbinafine nanoparticle was 78.52 nm. NP-TBH-PCL maintained a spherical form as NP-PCL. Both TEM results were smaller than the ones obtained from the DLS analyses, as expected, considering that each methodology uses a different technique and state (dry and in solution) to analyze the sample [24]. One can notice a background with smaller spheres which was probably caused by the formation of P407 micelles.

### 3.2. Drug Encapsulation Efficiency

Encapsulation efficiency (EE%) was performed and quantified using spectrophotometer UV. The EE of terbinafine nanoparticles was 98.81%. Hydrophobic drugs can be encapsulated into a PCL particle core due to the interactions with its hydrophobic chains. An example of this is the encapsulation efficiency of 96.1% of ketoconazole in PEG-PCL nanoparticles [25]. Terbinafine hydrochloride has a partition coefficient (LogP) of 5.51 (Drug Bank database) higher than ketoconazole that has LogP of 4.30 (PubChem Database). From log P information, we could infer that the hydrophobic interactions between PCL and terbinafine would be higher than PCL and ketoconazole, leading to a higher EE% of the nanoparticles. High EE% was also observed in PCL-NP stabilized with P407 [22,23].

### 3.3. Fourier Transform Infrared (FTIR)

**Figure 3:**
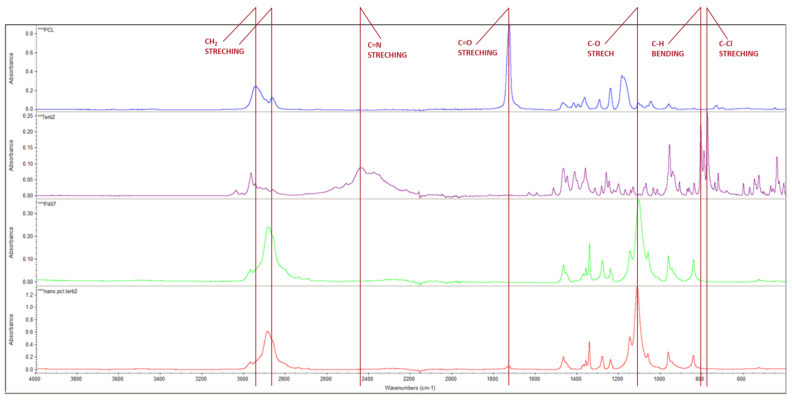
ATR-FTIR spectra of PCL, Terbinafine hydrochloride, P407, and the nanoparticle suspension with terbinafine, respectively.

The ATR-FTIR from the nanoparticles (Figure 3) showed a successful terbinafine encapsulation with the PCL nanoparticles. PCL shows a characteristic peak around 1728.09 cm^−1^ [carbonyl stretching], 2939.97 cm^−1^, 2855.85 cm^−1^ [asymmetric and symmetric CH_2_ stretching respectively], 1241.29 cm^−1^ [Asymmetric COC stretching] and 1186.97 cm^−1^ [OC=O stretching] [26]. The carbonyl stretching can also be seen in the nanoparticle spectra [1729.09 cm^−1^] with a low absorbance considering that the PCL spectra had a pure polymer and consequently a higher concentration than in the nanoparticle solution.

P407 show a characteristic peak around 1105.36 cm^−1^ [C-O stretch], and its presence in the nanoparticle spectra overlapped some of the other peaks at 2946.37cm^−1^ and 2867.44 cm^−1^ from PCL, with its own, 2885.37 cm^−1^.

Terbinafine hydrochloride shows peaks at 2442.76 cm^−1^ [C=N stretching], 1467.95 cm^−1^ [C-H bending], 807.97 cm^−1^ [CH bending], and 776.53 cm^−1^ [C-Cl stretching]. As the ATR-FTIR analyzes the surface of the particle and the medium that it is in [27], terbinafine hydrochloride should appear in the NP-TBH-PCL spectra, but when comparing the scale from the terbinafine with the one used in NP-TBH-PCL, it is possible that the terbinafine peak cannot be seen.

### 3.4. Terbinafine Release from Nanoparticle Formulation

**Figure 4:**
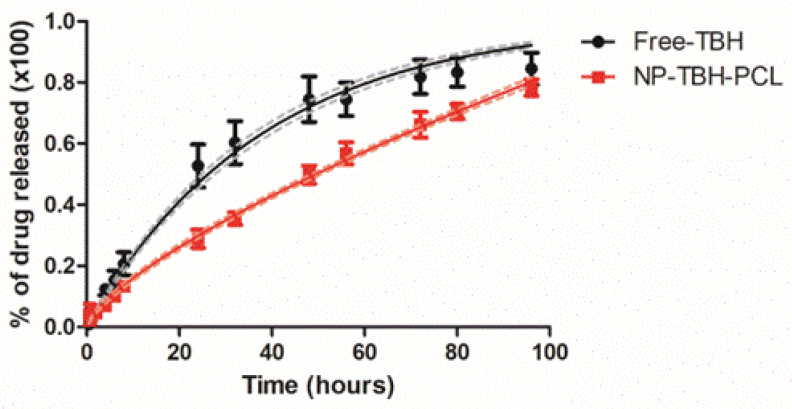
In vitro drug release profile of free-TBH and NP-TBH-PCL. Data is presented as average ± SD, n = 6. The continuous curves and their confidence interval (dashed lines) are the best mathematical fit.

The drug release profile in vitro was evaluated for free-TBH and NP-TBH-PCL for 96 h (Figure 4). In the first 6 h, there was an overlap between the free and encapsulated TBH, without significant differences (*p* > 0.05) between release percentage, which can be attributed to the diffusion of non-encapsulated TBH [28]. From 8 until 80 h, there are significant differences (*p* < 0.05 in 8 h and *p* < 0.001 in other measured times) between samples, and NP-TBH-PCL presented a lower release of TBH, proving that nanoencapsulation with PCL modified the release of TBH. Free-TBH and NP-TBH-PCL had a significant difference (*p* < 0.001) in permeation flux, which was 7.46 ± 0.18 and 6.43 ± 0.06 µg/cm^2^/h, respectively.

Mathematical models are a useful tool to fit the release data, helping to better understand data and to predict the release mechanism of a new dosage form [16,29]. After fitting the release profile curves (Table 3), it is possible to conclude that free-TBH presented a release following the first order model, while NP-TBH-PCL followed the Korsmeyer-Peppas model. Korsmeyer-Peppas is a model for predicting the release kinetics of polymeric systems [16], and was already used to describe drug release from NP-PCL [23]. The release type behavior can be indicated according to the release exponent (*n*). In the case of our nanoformulation, calculated *n* was 0.715 ± 0.014, indicating an anomalous transport release mechanism, where the diffusion and swelling/erosion of polymeric chains influenced TBH release [16,30,31].

### 3.5. Citotoxicity

**Figure 5:**
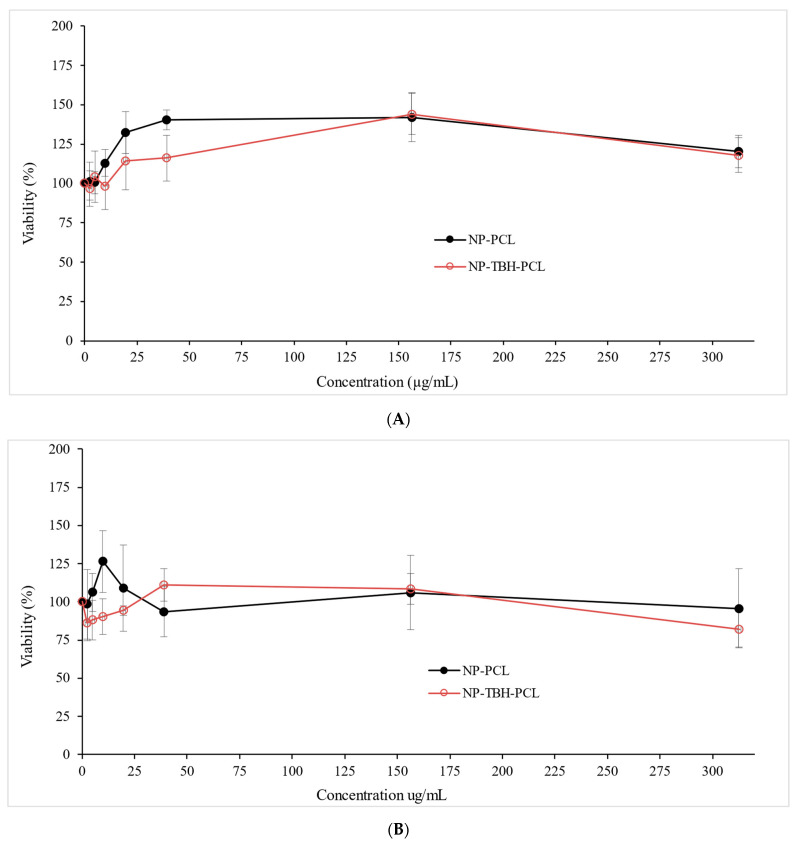
Viability after 24 h (**A**) and after 48 h (**B**) of NP-PCL and NP-TBH-PCL. Data are presented as average ± SD, n = 4).

The cytotoxicity of NP-PCL and NP-TBH-PCL was performed with HaCat cells (keratinocytes) by the MTT colorimetric assay after 24 and 48 h (Figure 5).

Even though at 24 h and 48 h NP-PCL viability was higher than NP-TBH-PCL in all tested concentrations, no significant difference (*p* < 0.05) was observed among treatments in the same concentration. The difference between treatments starts to diminish as the concentrations start to decrease. On the other hand, after 24 h treatment, significant sample concentration affects cell viability (*p* < 0.0001). Previous studies have also shown that NP-PCL did not affect HaCat viability, proving its biocompatibility [32,33]. Considering the standard deviation, it is possible to say that none of the concentrations showed less than 100% viability, and, on the contrary, in most of the concentrations it stimulated cell growth. In a previous study, Lim, et al. [34] developed a scaffold of PCL and blends of PCL and gelatin that demonstrated that human skin fibroblasts grew and proliferate well in those scaffolds, showing higher optical density than the control (plate without scaffold). When comparing 24 h with 48 h, some cell viability of less than 100% can be found, and lower than viability at 24 h. However, this decrease in cell viability comparing 24 h and 48 h treatments was significant only for NP-TBH-PCL at the highest tested concentration (*p* < 0.05), and was not significant in all other conditions (*p* > 0.05). The observation that NP-PCL shows higher viability than NP-TBH-PCL remains constant.

### 3.6. Nanohybrid Hydrogel Rheology

**Figure 6:**
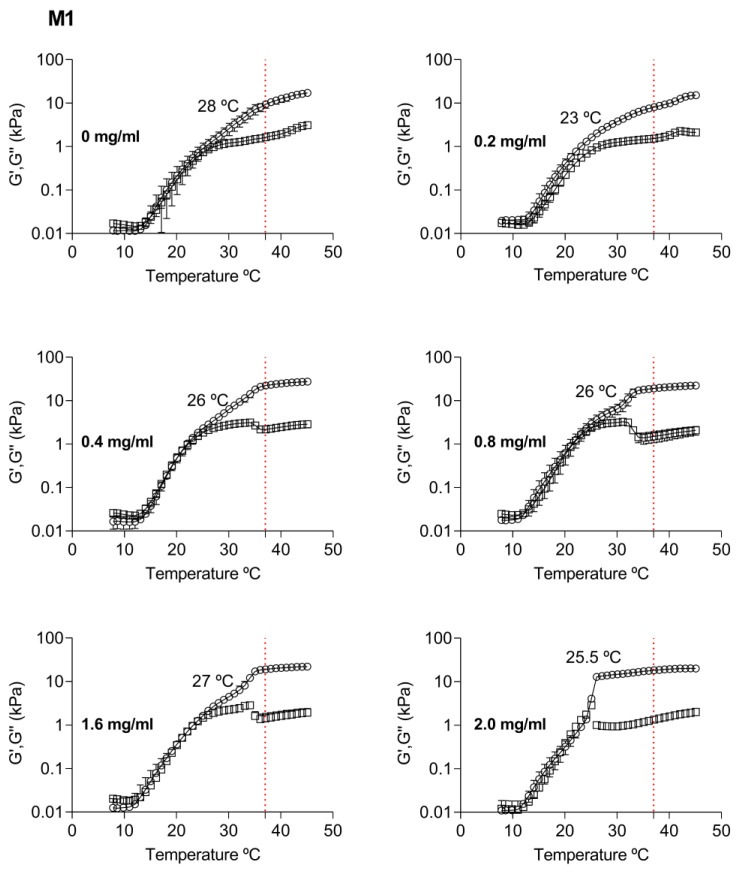
Rheological behavior of P407 18% Casein 10% hydrogel at different concentrations of the TBH-PCL nanoparticles which were added to the final P407 18% Casein 10% hydrogel (M1). Data are average ± SD (n = 4). In the vertical axis, the red dotted line refers to 37 °C, (○) storage modulus, and (□) loss modulus.

Hydrogel’s rheological behavior showed the influence of the NP-TBH-PCL addition order in the hydrogel mechanical properties, mainly in lower concentrations (Figure 6). At 37 °C in both preparation methods, the storage module (G′) was greater than the loss module (G″), i.e., the capacity of storage energy was improved when compared with the gel without nanoparticles, except to the composition of 0.2 mg in the M1.

For the first preparation method (M1), in the composition of 0.2 mg/mL, we noted a slight decrease in viscoelastic modules and a change from 28 to 23 °C (*p* < 0.05) in the gelation temperature. Based on the previous studies of poloxamer-based hydrogel [35], the changes in rheological behavior could be related to weaker hydrogen bonds occurring between hydrophilic groups of NP-TBH-PCL with the hydrophilic groups of the hydrogel. As the NP-TBH-PCL concentration increases, more hydrophilic groups are in the system interacting with the gel, which favors the gelation and viscoelastic modules. It was also well seen at concentrations above 0.4 mg/mL as the gelation temperature shifted from 28 to 26 °C (*p* < 0.05) and the viscoelastic modules were almost temperature-independent, showing solid gels (Figure 6). For this method, from 0.4 mg/mL, we can then obtain gels with enhanced mechanical properties.

**Figure 7:**
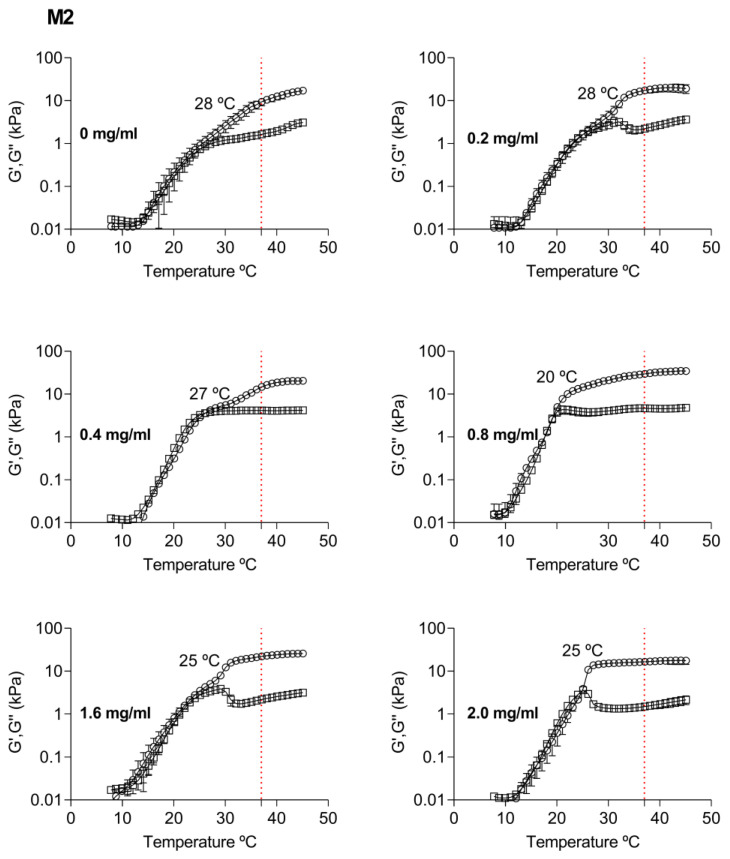
Rheological behavior of P407 18% casein 10% hydrogel at different concentrations of the TBH-PCL nanoparticles that were added to the casein solution, after which P407 was added into the solution (M2). Data are average ± SD (n = 4). In the vertical axis, a red dotted line refers to 37 °C, (○) storage modulus, and (□) loss modulus.

In the second method (M2), the gelation temperature also decreases from 28 to 26 °C (*p* < 0.05) with the incorporation of nanoparticles (Figure 7). To 0.8 mg/mL of nanoparticles, we noted a shift in the gelation temperature from 28 to 20 °C (*p* < 0.05). Moreover, we observed a significant increase in the storage module from 9.42 to 29.7 kPa (*p* < 0.05) at 37 °C. This behavior could be related to the nanoparticle’s addition order. The addition of poloxamer into a previous mixture of casein and NP-TBH-PCL can result in a quickly entangled chain that changes the viscoelastic behavior of the sample. In this method, 0.8 mg/mL is the concentration that showed more significant changes in the rheological behavior.

### 3.7. Nanohybrid Hydrogel Tixotropy

**Figure 8:**
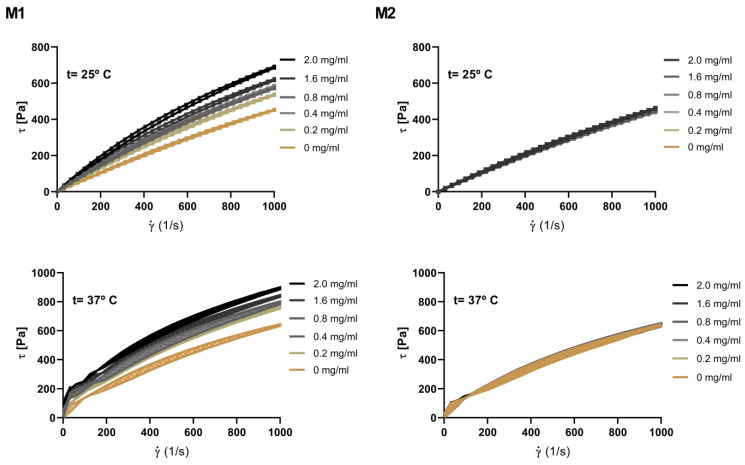
Hysteresis loop tests of P407 18% Casein 10% hydrogel at different concentrations of the TBH-PCL nanoparticles to (M1) and (M2) at 25 °C and 37 °C.

We performed hysteresis loop tests at 25 and 37 °C to evaluate the thixotropy of gel compositions. The hysteresis loop test systematically increases and decreases the shear rate between zero and a maximum value. A thixotropic sample will show a hysteresis loop. This enclosed area between curves estimates the degree of thixotropy resulting from the energy required to reorganize the material’s structure in response to mechanical stress [36]. Figure 8 shows the influence of NP-TBH-PCL addition order in hydrogel thixotropic properties. In both preparation methods, at 25 °C, the upward and downward cycles overlap independent of nanoparticle concentration. Therefore, the hysteresis area is not observed, indicating a time-independent behavior under shear load. On the other hand, when the temperature increases to 37 °C, a hysteresis loop area during shearing is observed. Then, at this temperature, the samples display thixotropic behavior, indicating that the structural strength decreases while shearing, but fully recovers after a certain period of rest, showing that their microstructure is susceptible to reversible alteration by moderate mechanical stress. This can lead to an improvement in the application of the product to the skin, since at body temperature there is a reduction in viscosity for a period.

### 3.8. Nanohybrid Hydrogel Spreadability

In water solutions, the amphiphilic character led the copolymers to self-aggregate into micelles, normally in nano-sized structures between 10 and 200 nm. Poloxamer water solutions exhibit a thermoreversible gelation, showing a sol-gel transition in physiological temperature (37 °C) [37], but with low adhesiveness, which can be improved by conjugation with other polymers [4]. The results presented in Table 4 show that the gel production process M1 presented less firmness and less adhesiveness (expressed by work of shear), when compared to M2. This indicates that the nanoparticles can interact with the micelles formed by the polymer and casein, altering the sol-gel transition that the gel undergoes when heated to 37 °C. Thus, for better spreadability, the M1 methodology is more effective.

### 3.9. Particle Release from Nanohybrid Hydrogel

The nanoparticles release kinetics from the hydrogel was assessed with NP-TBH-PCL and NP-PCL using PCL linked with fluorescein.

**Figure 9:**
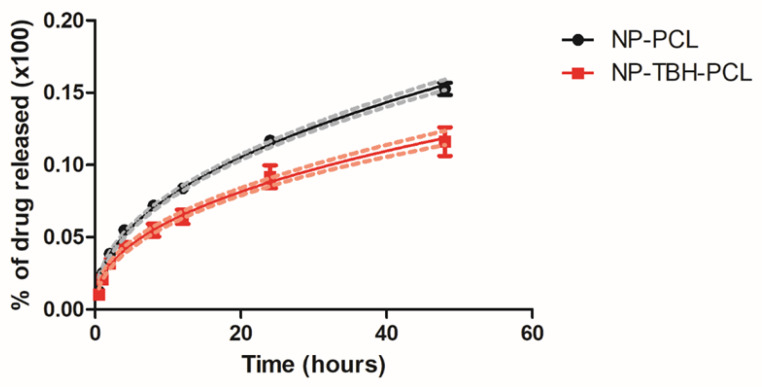
Cumulative release (%) of PCL-fluorescein-nanoparticle release with/out terbinafine from P407/casein hydrogel at 37 °C in artificial sweat at a pH of 4.7. Data are average ± SD (n = 4). Some error bars are obscured by the symbols due to their small size. The continuous curves and their confidence interval are the best mathematical fit (dashed lines).

In the absence of terbinafine, there was a higher release rate than with terbinafine (Figure 9). This difference is not significant *p* < 0.05. However, the release of both particles was slow if compared with the same hydrogel composition drug release described previously by [4]. This slow release could be justified by the PCL nanoparticle being inside the P407 micelles’ hydrophobic core, controlling its release. PCL is hydrophobic and its low affinity by the receptor medium together with a storage in the P407 core may have interfered with the release rate. This very slow release can be desired in long-term delivery systems in an implant or even a prothesis with drug release [38].

The release of both nanoparticles fitted the Kornsmeyer Peppas model, with an R^2^ of 0.9916 and 0.9743, a Kp of 0.028 ± 0.001, and 0.028 ± 0.001 for NP-PCL and NP-TBH-PCL, respectively. Furthermore, in both cases, the calculated release exponent (*n*) indicated that the NP release occurred by diffusion through the hydrogel matrix [31], with *n* values of 0.445 ± 0.009 and 0.429 ± 0.016 for NP-PCL and NP-TBH-PCL.

## 4. Conclusions

The nanoparticles with P407 as a stabilizer showed a low PDI and were stable over a month, together with high encapsulation efficiency, giving good evidence for further studies including ex vivo skin permeation and surface modulation. PCL nanoparticles successfully controlled the terbinafine release and NP’s did not interfere in the metabolic activity of the keratinocytes. The nanoparticles’ addition to the hydrogel significantly influenced the gelation temperature and the viscoelastic properties, and provided stable gels for the two preparation methods. Moreover, the nanoparticle addition order leads to different rheological and texture behaviors for the same nanoparticle concentration; thus, a suitable nanoparticle concentration to improve hydrogel mechanical properties can be addition order dependent. The hydrogels also showed a slow release of nanoparticles in the 48 h study, making them suitable for longer term evaluation. Further in vitro and in vivo studies should be carried out to prove the superiority of the developed system compared with conventional topical TBH treatments.

## Figures and Tables

**Table 1:** Settings and parameters of spreadability analysis using a TA.XT plus texturometer.

Settings and Parameters	Spreadability
Test Mode	Compression
Pre-Test Speed	1.00 mm/s
Test Speed	3.00 mm/s
Post-Test Speed	10.00 mm/s
Target Mode	Distance
Force	100.0 g
Distance	23.000 mm
Strain	10.0 %
Trigger Type	Button
Trigger Force	5.0 g
Probe	HDP/SR; spreadability rig
Temperature	37 °C

**Table 2:** The size distribution and Zeta potential of nanoparticles without and with terbinafine using the dynamic light-scattering (DLS) procedure employing Zetasizer.

		NP-PCL	NP-TBH-PCL
Size (nm)	Mean *	186.4 ± 0.4	196.7 ± 0.7
	D_(10)_	127.7 ± 8.1	139.0 ± 2.1
	D_(50)_ *	195.7 ± 3.1	205.0 ± 2.1
	D_(90)_	306.3 ± 29.5	305.0 ± 11.7
PDI *		0.093 ± 0.003	0.069 ± 0.010
Zeta potential (mV) *		−11.9 ± 0.7	−0.7 ± 0.1

Results presented as average ± SD of three measurements. NP-PCL = nanoparticles without terbinafine; NP-TBH-PCL = nanoparticles with terbinafine; D_(10)_ = 10% of detected particles that are equal to or smaller than this size; D_(50)_ = 50% of detected particles that are equal to or smaller than this size; D_(90)_ = 90% of detected particles that are equal to or smaller than this size; PDI = polydispersity index. The * sign shows parameters that present statistically significant differences by a Student’s *t*-test.

**Table 3:** Obtained kinetic parameters of the in vitro release profile of terbinafine.

	Free-TBH		NP-TBH-PCL	
	K_p_	R^2^	K_p_	R^2^
Zero Order	0.0114 ± 0.0004	0.7983	0.0091 ± 0.0001	0.9557
First Order	0.0265 ± 0.0007	0.9722	0.0149 ± 0.0002	0.9878
Higuchi model	0.0948 ± 0.0015	0.9459	0.0736 ± 0.0012	0.9518
Korsmeyer-Peppas model	0.0804 ± 0.0082	0.9483	0.0307 ± 0.0019	0.9903

Results presented as average ± SD, n = 6. Free-TBH = terbinafine solution; NP-TBH-PCL = nanoparticles with terbinafine; K_p_ = release rate constant; R^2^ = linear correlation coefficient.

**Table 4:** Firmness and work of shear of P407 18% Casein 10% hydrogel at different concentrations of the TBH-PCL nanoparticles added before (M1) and after (M2) gel formation.

Formulation		Firmness (g)	Work of Shear (g.s)
Method	NP Concentration (mg/mL)		
Blank	-	295.13 ± 54.76 ^a^	212.74 ± 55.54 ^a^
M1	0.2	255.35 ± 30.42 ^a,d,e,f,g^	255.35 ± 30.42 ^a,d,e,f,g^
	0.4	262.21 ± 42.72 ^a^	262.21 ± 42.72 ^a^
	0.8	223.79 ± 53.75 ^a,e,f,g^	223.79 ± 53.75 ^a,e,f,g^
	1.6	219.42 ± 52.90 ^a^	219.42 ± 52.90 ^a^
	2.0	354.03 ± 23.41 ^a,b^	354.03 ± 23.41 ^a,b^
M2	0.2	395.50 ± 27.00 ^b^	395.50 ± 27.00 ^b^
	0.4	499.48 ± 45.54 ^b^	499.48 ± 45.54 ^b^
	0.8	322.43 ± 22.77 ^a,b,c,d^	322.43 ± 22.77 ^a,b,c,d^
	1.6	453.18 ± 58.92 ^b^	453.18 ± 58.92 ^b^
	2.0	365.03 ± 26.11 ^a,b,c^	365.03 ± 26.11 ^a,b,c^

The results are presented as average ± SD (n = 3). Different letters in the same column indicate significant differences between samples according to one-way ANOVA (*p* < 0.05) and Tukey’s test.

## Data Availability

The data presented in this study are available upon request from the corresponding author.
